# Development of a nonhuman primate model for mammalian bornavirus infection

**DOI:** 10.1093/pnasnexus/pgac073

**Published:** 2022-06-08

**Authors:** Kore Schlottau, Friederike Feldmann, Patrick W Hanley, Jamie Lovaglio, Tsing-Lee Tang-Huau, Kimberly Meade-White, Julie Callison, Brandi N Williamson, Rebecca Rosenke, Dan Long, Claudia Wylezich, Dirk Höper, Christiane Herden, Dana Scott, Donata Hoffmann, Greg Saturday, Martin Beer, Heinz Feldmann

**Affiliations:** Institute of Diagnostic Virology, Friedrich-Loeffler-Institut, Südufer 10, 17493 Greifswald-Insel Riems, Germany; Rocky Mountain Veterinary Branch, Division of Intramural Research, National Institute of Allergy and Infectious Diseases, National Institutes of Health, Hamilton, MT 59840, USA; Rocky Mountain Veterinary Branch, Division of Intramural Research, National Institute of Allergy and Infectious Diseases, National Institutes of Health, Hamilton, MT 59840, USA; Rocky Mountain Veterinary Branch, Division of Intramural Research, National Institute of Allergy and Infectious Diseases, National Institutes of Health, Hamilton, MT 59840, USA; Laboratory of Virology, Division of Intramural Research, National Institute of Allergy and Infectious Diseases, National Institutes of Health, Hamilton, MT 59840, USA; Laboratory of Virology, Division of Intramural Research, National Institute of Allergy and Infectious Diseases, National Institutes of Health, Hamilton, MT 59840, USA; Laboratory of Virology, Division of Intramural Research, National Institute of Allergy and Infectious Diseases, National Institutes of Health, Hamilton, MT 59840, USA; Laboratory of Virology, Division of Intramural Research, National Institute of Allergy and Infectious Diseases, National Institutes of Health, Hamilton, MT 59840, USA; Rocky Mountain Veterinary Branch, Division of Intramural Research, National Institute of Allergy and Infectious Diseases, National Institutes of Health, Hamilton, MT 59840, USA; Rocky Mountain Veterinary Branch, Division of Intramural Research, National Institute of Allergy and Infectious Diseases, National Institutes of Health, Hamilton, MT 59840, USA; Institute of Diagnostic Virology, Friedrich-Loeffler-Institut, Südufer 10, 17493 Greifswald-Insel Riems, Germany; Institute of Diagnostic Virology, Friedrich-Loeffler-Institut, Südufer 10, 17493 Greifswald-Insel Riems, Germany; Justus-Liebig-Universität, Institute of Veterinary Pathology, 35390 Gießen, Germany; Rocky Mountain Veterinary Branch, Division of Intramural Research, National Institute of Allergy and Infectious Diseases, National Institutes of Health, Hamilton, MT 59840, USA; Institute of Diagnostic Virology, Friedrich-Loeffler-Institut, Südufer 10, 17493 Greifswald-Insel Riems, Germany; Rocky Mountain Veterinary Branch, Division of Intramural Research, National Institute of Allergy and Infectious Diseases, National Institutes of Health, Hamilton, MT 59840, USA; Institute of Diagnostic Virology, Friedrich-Loeffler-Institut, Südufer 10, 17493 Greifswald-Insel Riems, Germany; Laboratory of Virology, Division of Intramural Research, National Institute of Allergy and Infectious Diseases, National Institutes of Health, Hamilton, MT 59840, USA

**Keywords:** bornavirus, nonhuman primate, animal model

## Abstract

Until recently, it was assumed that members of the family *Bornaviridae* could not induce severe disease in humans. Today, however, Borna disease virus 1 (BoDV-1), as well as the more recently emerged variegated squirrel bornavirus 1 (VSBV-1), are known as causative agents of lethal encephalitis in humans. In order to establish animal models reflecting the pathogenesis in humans and for countermeasure efficacy testing, we infected twelve rhesus macaques (*Macaca mulatta)* either with VSBV-1 or with BoDV-1. For each virus, three monkeys each were inoculated with 2 × 10^4^ focus forming units by the intracerebral route or by multiple peripheral routes (intranasal, conjunctival, intramuscular, and subcutaneous; same dose in total). All BoDV-1 and VSBV-1 intracerebrally infected monkeys developed severe neurological signs around 5 to 6 or 8 to 12 weeks postinfection, respectively. Focal myoclonus and tremors were the most prominent observations in BoDV-1 and VSBV-1-infected animals. VSBV-1-infected animals also showed behavioral changes. Only one BoDV-1 peripherally infected animal developed similar disease manifestations. All animals with severe clinical disease showed high viral loads in brain tissues and displayed perivascular mononuclear cuffs with a predominance of lymphocytes and similar meningeal inflammatory infiltrates. In summary, rhesus macaques intracerebrally infected with mammalian bornaviruses develop a human-like disease and may serve as surrogate models for human bornavirus infection.

Significance Statement Since the zoonotic potential of mammalian bornaviruses has been demonstrated in recent years, human cases are increasing in the endemic areas. Overall, the disease caused by Borna disease virus 1 (BoDV-1) or variegated squirrel bornavirus 1 (VSBV-1) occurs very rarely, but unfortunately most of the infected patients develop fatal encephalitis. Not much is known regarding transmission and incubation period, let alone that no antivirals or therapeutics have been tested. Therefore, a surrogate animal model would be urgently needed to fill this knowledge gap. In this study, we infected rhesus macaques with two different mammalian bornaviruses by different routes and established a surrogate animal model for bornavirus caused encephalitis.

## Introduction

The variegated squirrel bornavirus 1 (VSBV-1) was first isolated from brain tissue of a variegated squirrel (subfamily *Sciurinae*) and three fatally infected squirrel breeders in Germany (1). Subsequently, squirrels of the genus *Callosciurus* (subfamily *Callosciurinae*) were also shown to be infected and likely represent another reservoir host for this emerging pathogen (2, 3). Meanwhile, VSBV-1 infection has also been associated with the death of two animal caretakers (4, 5) and symptomatic infection of another squirrel breeder (6). In total of six human VSBV-1 cases have been confirmed in Germany. So far, there are no reports from other countries

VSBV-1 belongs to the family *Bornaviridae* within the order *Mononegavirales* and forms the new species *Orthobornavirus sciuri*, which shares less than 75% nucleotide sequence homology with other bornaviruses (7). Today, there is still very little knowledge on epidemiology, transmission, and pathogenesis of VSBV-1. Autopsies of human VSBV-1 cases have shown inflammatory lesions in brain areas positive for VSBV-1 genome and antigen consisting mainly of CD4+ and CD8+ T cells and perivascular B cells (8).

The closest relative of VSBV-1 is the “classical” Borna disease virus 1 (BoDV-1), which belongs to the species *Orthobornavirus bornaense* (9) and mainly infects horses and sheep resulting in fatal meningoencephalitis (10, 11). The reservoir host of BoDV-1 is the bicolored white-toothed shrew, which is endemic in central Europe (12, 13). The exact transmission routes within the reservoir species and to dead-end hosts are unknown. Infected shrews shed virus in feces and urine. The zoonotic potential of BoDV-1 was heavily investigated and controversially discussed in the past but was finally rejected by several research groups (14–16). In 2018, however, BoDV-1 infection was unequivocally confirmed as the cause of lethal encephalitis in a cluster of solid organ transplant recipients (17). Furthermore, BoDV-1 caused fatal encephalitis in a young student (18) and retrospective studies revealed an additional 35 positive BoDV-1 cases in the endemic region (5, 19–22). So far, nearly 40 human BoDV-1 cases were reported in Germany with no reports from neighboring countries (Austria, Liechtenstein and Switzerland). Except for the iatrogenic transmission of BoDV-1 through organ transplantation, transmission from a dead-end host has not been reported.

BoDV-1 has a long research history and several small animal models have been investigated with the Lewis rat being the best-established model (11, 23, 24). To date, VSBV-1 infection studies have been conducted in Lewis rats (25), but not in nonhuman primates (NHPs). Several decades ago, two research groups infected NHPs with BoDV-1 by the intracerebral route resulting in severe neurological signs 4 to 8 weeks after infection, but detailed further investigations into viral tissue distribution and route of infection were not performed (26, 27). No further work has been published utilizing the NHP model, likely due to the fact that BoDV-1 was not considered a human pathogen. Now, given the recently demonstrated zoonotic potential of certain bornaviruses including BoDV-1, a NHP disease model would be of tremendous importance for pathogenesis studies and countermeasure development.

Here, we infected rhesus macaques with VSBV-1 and BoDV-1 by either the intracerebral or a combination of peripheral routes. Animals were monitored for disease progression and outcome. All intracerebrally infected animals were euthanized due to severe meningoencephalitis and demonstrated high viral loads in brain tissues. In contrast, infection by the peripheral route caused disease in at least one animal demonstrating the possibility and likewise inconsistency of this exposure route to cause infection. Altogether, we established the rhesus macaque as a surrogate disease model for human bornavirus infections. This model could provide deeper insights into transmissions pathways, pathogenesis, and countermeasure development.

## Results

### Clinical observations

**Fig. 1.**
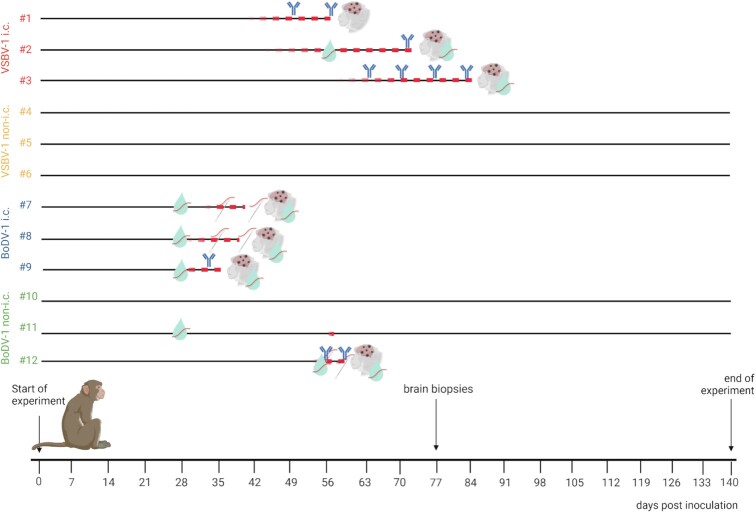
Experimental schedule and time of viral RNA and antibody detection. Red dashed lines depict the time period of clinical signs for each individual animal. Viral antigen was found in brain samples from all seven diseased NHPs (brain graphic). Viral RNA was found in CSF (turquoise drop) and oral swab samples. Bornavirus reactive antibodies were found in five of the infected NHPs. Key: i.c. = intracerebral; non-i.c. = peripheral routes. Created with BioRender.com.

Table 1 and Figure 1 provide information on study design and outcome. Significant changes in body weight and body temperature as well as hematology and blood chemistry parameters were not noted throughout the study (Figure S1, Supplementary Material). Clinical scores are shown in Figure S2 (Supplementary Material).

**Table 1.** Study design and outcome of bornavirus infection experiments in rhesus macaques.

**Group ID**	**Virus**	**Inoculation**	A**nimal number**	**Sex**	**Day of necropsy**	T**issue bornavirus RNA positive**	**Bornavirus reactive antibodies**	**Histological changes related to bornaviruses**
**Group 1**	VSBV-1	Intracerebral	#1	Male	56	Yes	Yes	Yes
			#2	Female	71	Yes	Yes	Yes
			#3	Male	84	Yes	Yes	Yes
**Group 2**	VSBV-1	Peripheral	#4	Female	140	No	No	No
			#5	Male	140	No	No	No
			#6	Female	140	No	No	No
**Group 3**	BoDV-1	Intracerebral	#7	Male	41	Yes	No	Yes
			#8	Female	40	Yes	No	Yes
			#9	Male	35	Yes	Yes	Yes
**Group 4**	BoDV-1	Peripheral	#10	Male	140	No	No	No
			#11	Male	140	No	No	No
			#12	Female	59	Yes	Yes	Yes

Peripheral: (intranasal, conjunctival, subcutaneous, and intramuscular).

### VSBV-1-inoculated animals

Initial clinical signs of disease were noted in all intracerebrally VSBV-1 inoculated animals between 36 – 47 days post-inoculation (dpi) and included anorexia (loss of appetite), mild myoclonus (involuntary muscle twitching) of the extremities, somnolence (sleepiness), and decreased activity. Following these initial signs, clinical presentation varied finally resulting in severe disease accompanied by behavioral changes such as abnormal eating disorders (NHP #1 and NHP #2), increased agitation, and aggression (NHP #2 and NHP #3). A total of two of the three animals presented with partial paralysis of the extremities and had to be euthanatized (71 and 84 dpi, NHP #2 and NHP #3, respectively). One animal had an acute respiratory arrest immediately following CSF collection on 28 dpi and was resuscitated. At 56 dpi, this same animal did not recover from anesthesia and was promptly euthanized (NHP #1). All VSBV-1 peripherally inoculated animals were asymptomatic throughout the entire study.

### BoDV-1-inoculated animals

Initial clinical signs of disease were noted in two of three intracerebrally BoDV-1 inoculated animals starting at 28 dpi including anorexia, piloerection (goose bumps) and decreased activity (NHP #8 and NHP #9). Clinical signs progressed to a severe state that included myoclonus (involuntary muscle twitching) in the extremities, ataxia (uncoordinated muscle movements), excessive blepharospasm (inability to open eyelid), significant anorexia, and lethargy. The two animals were euthanized either on 35 (NHP #9) and 40 dpi (NHP #8). The other animal started to show clinical signs on 36 dpi with anorexia, myoclonus in the extremities, and tremors (involuntary muscle contractions). At 41 dpi, the animal became ataxic and had to be euthanized (NHP #7). One of the peripherally BoDV-1-inoculated animals remained asymptomatic throughout the entire study (NHP #10). Another animal showed mild neurological signs with myoclonus of the extremities at 58 dpi but fully recovered thereafter (NHP #11). The third animal (NHP #12) displayed initial clinical signs on 54 dpi including ataxia, decreased activity, and anorexia. These clinical signs progressed to partial paralysis, tremors, and somnolence by 59 dpi when the animal had to be euthanized.

### Genome detection

Viral shedding from mucosal membranes was monitored by analyzing oral, nasal, and rectal swabs weekly. Furthermore, blood samples and CSF were drawn on a weekly or monthly basis, respectively. Results are displayed in Table 2. No VSBV-1 genome could be detected in swabs, but two rhesus macaques from group 1 had detectable amounts of VSBV-1 RNA in their CSF samples (NHP #2 at 56 and 71 dpi; NHP #3 at 84 dpi; and between 1.07 × 10^6^ and 1.79 × 10^4^ copies per ml, respectively). NHP #3 was the only animal out of six remaining animals at 77 dpi, with a VSBV-1 RNA-positive brain biopsy. BoDV-1 RNA could be detected in oral swabs from two animals in group 3 (NHP #7 at 35 and 41 dpi; NHP # 8 at 35, and 40 dpi, genome copies per ml ranging from 1.32 × 10^3^ to 3.43 × 10^4^) and one animal in group 4 (NHP #12 at 56 and 59 dpi; 3.66 × 10^1^ and 4.04 × 10^4^ copies per ml). These three animals also had BoDV-1 RNA-positive CSF samples (NHP #7 at 28 and 41 dpi; NHP #8 at 28 and 40 dpi; and NHP #12 at 56 and 59 dpi with copies per ml between 6.23 × 10^1^ and 1.97 × 10^7^). CSF samples were also BoDV-1 RNA-positive for NHP #9 (28 and 35 dpi) and NHP #11 (28 dpi; 5.42 × 10° copies per ml). In addition, BoDV-1 RNA could be detected in the urine of NHP #12 at day of necropsy (7.38 × 10^6^ copies per ml). All blood and nasal and rectal swab samples were BoDV-1 RNA negative (Table 2). Overall, only animals exhibiting signs of disease showed positive results for bornavirus RNA.

**Table 2.** Genome detection and serology test results on clinical specimens.

**(A)**	**VSBV-1 RT-qPCR**						**BoDV-1 RT-qPCR**					
**Animal ID**	**#1 (i.c.)**	**#2(i.c.)**	**#3(i.c.)**	**#4 (non-i.c.)**	**#5 (non-i.c.)**	**#6 (non- i-.c.)**	**#7(i.c.)**	**#8(i.c.)**	**#9(i.c.)**	**#10 (non-i.c.)**	**#11 (non-i.c.)**	**#12 (non-i.c.)**
**Blood**	–	–	–	–	–	–	–	–	–	–	–	–
**Oral swab**	–	–	–	–	–	–	35 dpi (37.75), 41 dpi (32.43)	35 dpi (33.26), 40 dpi (30.85)	–	–	–	56 dpi (36.58), 59 dpi (34.57)
**Nasal swab**	–	–	–	–	–	–	–	–	–	–	–	–
**Rectal swab**	–	–	–	–	–	–		–	–	–	–	–
**CSF**	–	56 dpi (33.49), 71 dpi (20.87)	84 dpi (36.61)	–	–	–	28 dpi (32.87), 41 dpi (29.04)	28 dpi (18.25), 40 dpi (33.21)	28 dpi (35.34), 35 dpi (33.83)	–	28 dpi (39.89)	56 dpi (36.35), 59 dpi (22.89)
**Brain biopsy**	n.d.	n.d.	77 dpi (27.98)	–	–	–	n.d.	n.d.	n.d.	–	–	n.d.
**Urine**												59 dpi (24.72)
**(B)**	**Bornavirus-reactive antibodies**											
**Animal ID**	**#1 (i.c.)**	**#2** **(i.c.)**	**#3** **(i.c.)**	**#4 (non-i.c.)**	**#5 (non-i.c.)**	**#6 (non- i-.c.)**	**#7** **(i.c.)**	**#8** **(i.c.)**	**#9** **(i.c.)**	**#10 (non-i.c.)**	**#11 (non-i.c.)**	**#12 (non-i.c.)**
**Sera**	49 dpi (1:80); 56 dpi (1:640)	70 dpi (1:320)	63 dpi (1:160); 70 dpi (1:320); 77 dpi (1:640); 84 dpi (1:1280)	–	–	–	–	–	35 dpi (1:80)	–	–	56 dpi (1:80); 59 dpi (1:2560)
**CSF**	–	–	84 dpi (1:40)	–	–	–	–	–	–	–	–	–

(A) RT-qPCR results: blood and swabs were tested weekly and at necropsy, CSF monthly and at necropsy, urine only at necropsy, and brain biopsies were taken at day 77/78 postinfection. Cq values are given for time points with RT-qPCR positive results in brackets. (B) Serology results: sera samples were tested weekly and CSF monthly. Titres are given for time points with detectable antibodies.—= RT-qPCR negative or antibodies below the cut off (< 1:16) on all dates; n.d. = dot done. i.c. = intracerebral; and non-i.c. = peripheral route.

**Fig. 2.**
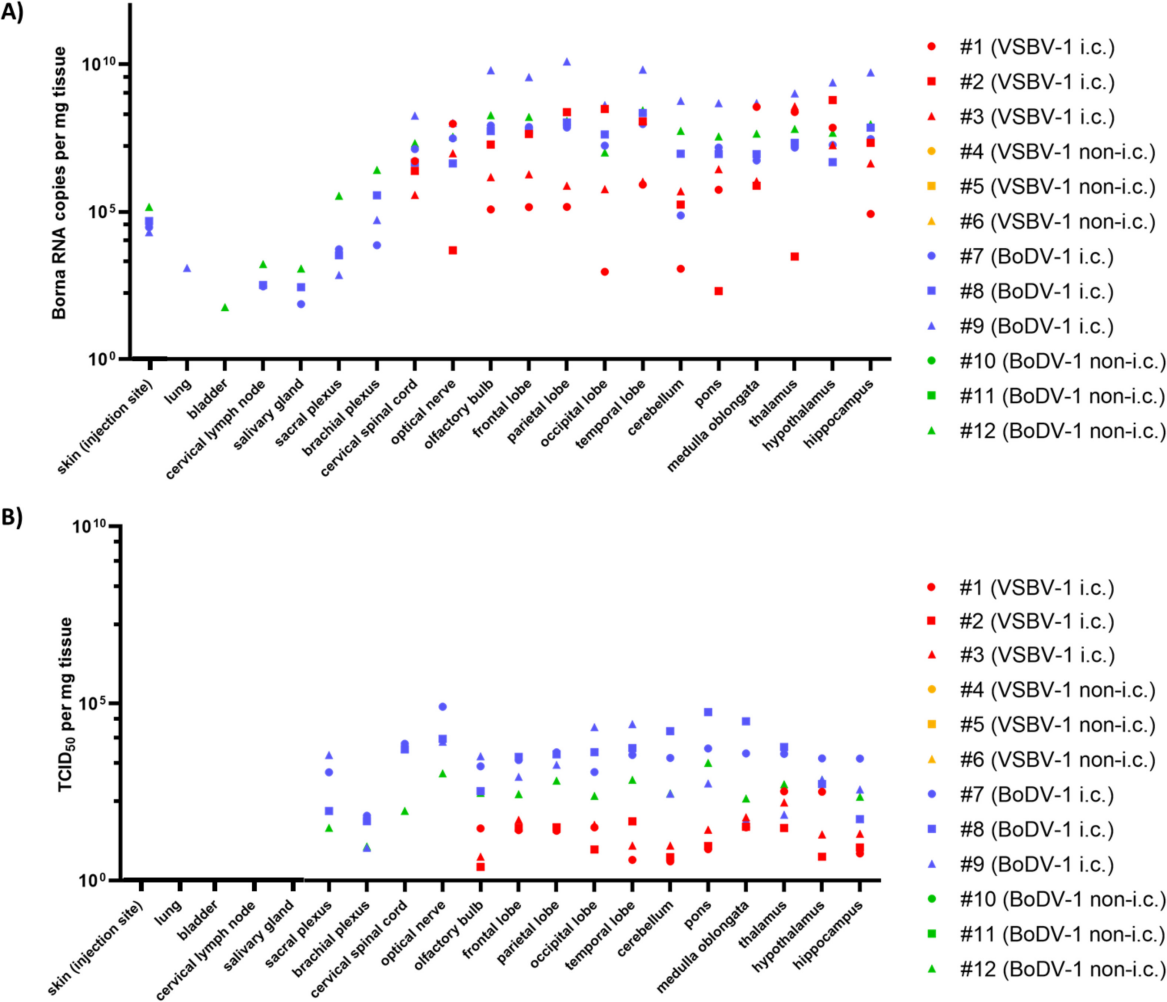
BoDV-1 (#7–12) and VSBV-1 (#1–6) genome load (A) and infectious titers (B) in selected organs derived from the individual NHPs. i.c. = intracerebral; non-i.c. = peripheral routes per mg tissue.

At necropsy, all tissues analyzed for group 2 (VSBV-1 peripherally inoculated) were VSBV-1 RNA negative, whereas all brain samples from group 1 (VSBV-1 intracerebrally inoculated) were positive for VSBV-1 RNA (Figure 2A). In detail, for NHP #1 genome copies per mg brain tissue varied between 9.18 × 10^2^ (occipital lobe) and 3.60 × 10^8^ (medulla oblongata), for NHP #2 between 2.04 × 10^2^ (pons) and 6.08 × 10^8^ (hypothalamus) and for NHP #3 between 4.98 × 10^5^ (cerebellum) and 3.81 × 10^8^ (thalamus). BoDV-1 RNA was detectable in tissue samples from four out of six BoDV-1-inoculated animals (Figure 2A). All three animals in group 3 (BoDV-1 intracerebrally inoculated) and one animal from group 4 (BoDV-1 peripherally inoculated) had detectable BoDV-1 genome copy numbers per mg brain tissue. In detail, genome copies for NHP #7 varied between 7.48 × 10^4^ (cerebellum) and 8.37 × 10^7^ (frontal lobe), for NHP #8 between 4.85 × 10^6^ (hypothalamus) and 2.22 × 10^8^ (temporal lobe), for NHP #9 between 4.15 × 10^8^ (occipital lobe) and 1.24 × 10^10^ (parietal lobe), and for NHP #12 between 1.03 × 10^7^ (occipital lobe) and 2.76 × 10^8^ (temporal lobe). Moreover, with these four animals BoDV-1 RNA could also be detected in the sacral and brachial plexus as well as in skin samples from the inoculation site (between 7.17 × 10^2^ and 2.64 × 10^6^ copies per mg tissue). Cervical lymph node and salivary gland were BoDV-1 RNA positive in NHPs #7, #8, and #12 (between 7.44 × 10^1^ and 1.70 × 10^3^ copies per mg tissue). In addition, lung tissue was BoDV-1 RNA-positive at a lower level for NHP #9 (1.24 × 10^3^ genome copies per mg tissue) and bladder tissue for NHP #12 (5.85 × 10^1^ genome copies per mg tissue).

Comparison of viral genome sequences from the brain samples to the inocula revealed no mutations for the BoDV-1-infected animals. In the three VSBV-1 intracerebrally infected NHPs, two amino acid changes occurred in the polymerase gene (N102D and N1564S).

### Infectious virus

All swab, blood, and urine samples were negative for infectious bornavirus. VSVB-1 and BoDV-1 could be isolated from brain tissue derived from multiple locations as well as some CSF samples (Figure 2B). All other tissue samples were negative for infectious virus despite detectable bornaviral RNA. In detail, for the VSBV-1 intracerebrally infected animals, viral titres varied for NHP #1 between 10^0.5^ (cerebellum) and 10^2.6^ (thalamus), for NHP #2 between 10^0.7^ (cerebellum) and 10^1.5^ (medulla oblongata), and for NHP #3 between 10^0.6^ (olfactory bulb) and 10^2.2^ (thalamus). The BoDV-1 intracerebrally infected rhesus macaques had generally higher viral titres and ranged for NHP #7 from 10^1.8^ (brachial plexus) to 10^4.9^ (optical nerve), for NHP #8 from 10^1.7^ (brachial plexus) to 10^4.8^ (pons), for NHP #9 from 10^0.9^ (brachial plexus) to 10^4.4^ (temporal lobe), and for NHP #12 from 10^1^ (brachial plexus) to 10^4^ (optical nerve). Terminal CSF samples for NHPs #7, #8, #9, and #12 contained infectious BoDV-1 with 10^1.5^ TCID_50_ per ml.

### Pathology and detection of viral antigen by immunohistochemistry

**Fig. 3.**
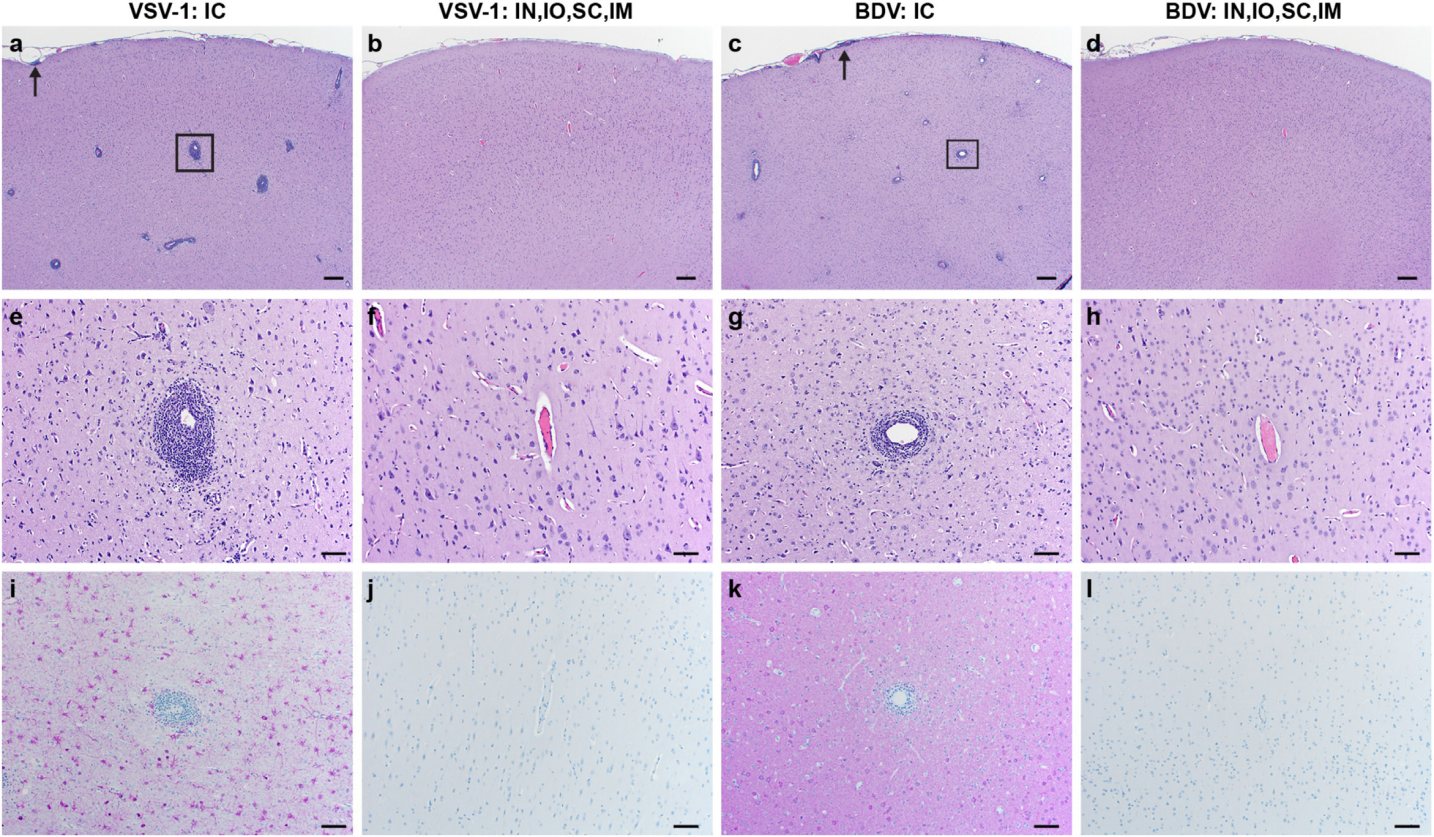
Histopathology and immunohistochemistry on brain tissues derived from NHPs infected with VSBV-1 and BoDV-1. (a)–(d): H&E 20X; bar = 200 µm, a/c: lymphocytic perivascular cuffing (box), and meningeal infiltrates (arrows), (b)/(d) = no histologic lesions. (e)–(h): H&E 100X; bar = 50 µm, e/g: prominent lymphocytic perivascular cuffing, and f/h: no perivascular cuffing. (i)–(l): IHC 100X; bar = 50 µm, (i)/(k): diffuse immunoreactive neurons, astrocytes, and microglia, (j)/(l): no immunoreactivity. I.C. = intracerebral; IN, IO, SC, and IM = peripheral routes.

All animals in the intracerebrally inoculated groups had mild to marked perivascular leucocyte cuffs with a predominance of lymphocytes throughout the brainstem and cerebrum and minimal to moderate meningeal infiltrates. Only two animals in the peripherally inoculated groups showed minimal to mild mononuclear perivascular cuffing (NHP #6 and NHP #12, respectively) and one animal (NHP #12) had minimal mononuclear meningeal infiltrates (see Figure 3 and Table S2, Supplementary Material). Leukomalacia and Gitter cell accumulation were identified in four of six animals in the intracerebrally inoculated animals (groups 1 and 3) and in three of six animals in the peripherally inoculated animals (groups 2 and 4). Wallerian degeneration of the spinal cord was identified in all peripherally inoculated animals but only one animal in the intracerebrally inoculated NHPs (NHP #1). Wallerian degeneration within the brain stem could be shown in NHP #1 and NHP #8 (Table S2, Supplementary Material). In addition to the lesions in the central nervous system, four animals demonstrated histologic changes in the peripheral nervous system (Figure S3, Supplementary Material). NHP #7 and NHP #8 had small lymphocytic inflammatory infiltrates in the brachial plexus and NHP #1 and NHP #3 had similar inflammatory infiltrates in the optic nerve. All other lesions noted in the remaining tissues are incidental findings and are not considered to be clinically significant (Table S2, Supplementary Material).

Immunohistochemistry (IHC) revealed moderate to strong immunoreactivity in brain tissue for VSBV-1 and BoDV-1 intracerebrally inoculated animals (groups 1 and 3, respectively). Immunoreactivity was not detected in any of the peripherally inoculated animals (groups 2 and 4) except for NHP #12. NHP #12 displayed very strong immunoreactivity in the brain for BoDV-1 compared to the other samples. IHC on cervical spinal cords were similar to the brain results except for NHP #3 that demonstrated no immunoreactivity (Table S3, Supplementary Material). IHC was performed on additional tissues from NHPs #7, #8, #9, and #12 to confirm positive PCR data. Brachial plexus, skin, urinary bladder, cervical lymph node, and salivary gland all had associated nerves that were immunoreactive for BoDV-1 (Figure S2, Supplementary Material).

**Fig. 4.**
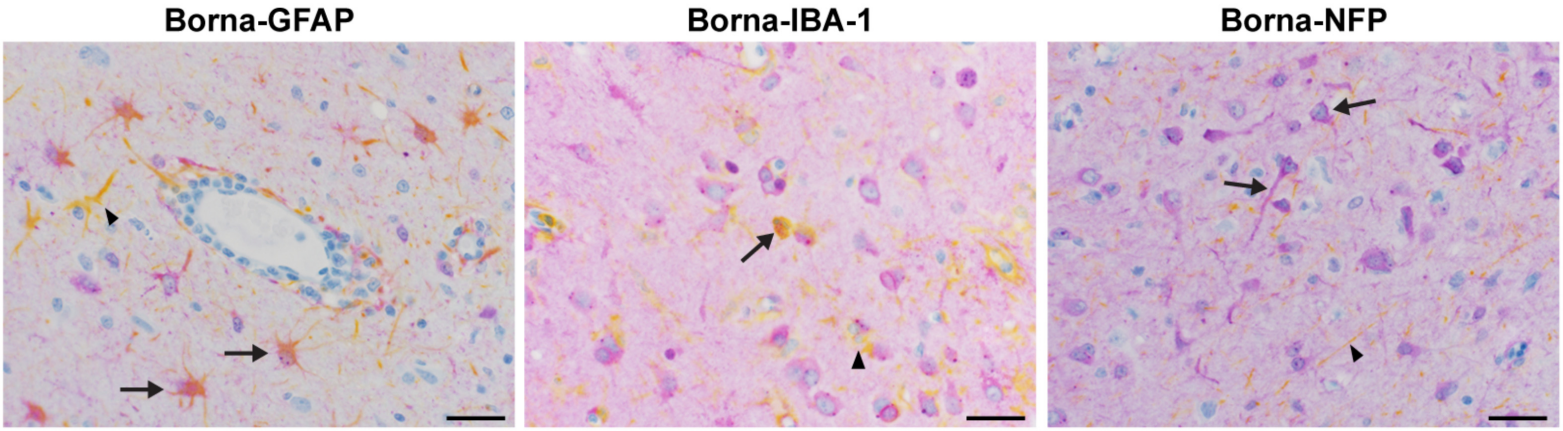
Double staining with cross-reactive BoDV-1 P antibody and GFAP, IBA1, and NFP. 100X; bar = 20 µm. Bornavirus positive cells are purple. GFAP: yellow cells are astrocytes (arrowhead); IBA1: yellow cells are microglia (arrowhead); and NFP: yellow cells are nerve axons (arrowhead). Dual staining is colored orange/pink (arrows).

Double staining using a cross-reactive bornavirus phosphoprotein antibody and GFAP (astrocytes) and NFP (neurons) revealed both cell types were immunoreactive for bornavirus. Rare dual immunoreactivity was identified in microglia utilizing Iba-1 (Figure 4).

### Antibody detection and T-cell response

The three VSBV-1 intracerebrally inoculated NHPs in group 1 showed bornavirus-reactive IgG antibody titers, whereas no antibodies could be detected in group 2 animals that were peripherally inoculated. For NHP #1, antibodies were initially detected at 49 dpi (titer 1/80) and increased in titer at 56 dpi (1/640). For NHP #2, only sera taken at 71 dpi contained bornavirus-specific antibodies (titer 1/320). NHP #3 was initially IgG-positive at 63 dpi and reached the highest titer at 84 dpi (1/1280). This was the only animal with an antibody-positive CSF sample (84 dpi, titer 1/40). Interestingly, in the BoDV-1-inoculated groups seroconversion occurred only with one animal each in group 3 (NHP #9, 35 dpi, titer 1/80) and group 4 (NHP #12, 56, and 59 dpi, highest reactive serum dilution 1/80 and 1/2560, respectively; see Table 2B).

T cell responses specific to VSBV-1 and BoDV-1 were assessed by an interferon-γ (IFN-γ) and TNF-α ELISpot assay on peripheral blood mononuclear cells (PBMCs) isolated at necropsy. No T-cell responses to bornaviruses could be detected for a multiplicity of infection between 0.01 and 1.

## Discussion

Our study focused on the disease-causing potential of VSBV-1 and BoDV-1 in an NHP model, the rhesus macaques, inoculated either by the intracerebral or a combination of possible naturally occurring peripheral (intranasal, intramuscular, conjunctival, and subcutaneous) routes of infection. The main goal was to establish an animal model reflecting human-like disease. Animal disease models are of high importance since the zoonotic capacity of bornaviruses has been established causing lethal encephalitis in humans (28). For BoDV-1, adult Lewis rats are used to partially mimic the accidental dead-end host infection, but NHPs are assumed to be a more suitable animal model due to the close evolutionary relationship to humans. Especially for VSBV-1, a suitable model is not available (23–25) but urgently needed.

Rhesus macaques were selected here to establish a disease model for the emerging VSBV-1 and compare it to BoDV-1 infection for which limited NHP studies were performed several decades ago (26, 27). In our trial, all intracerebrally infected animals developed severe encephalitis with multiple neurologic signs within 4 to 5 weeks leading to euthanasia within 4 to 6 days after onset of disease (Figure 1 and Figure S1, Supplementary Material). The incubation period matched the data published by Stitz et al. (26) with intracerebrally BoDV-1-infected rhesus macaques developing encephalitis after 4 to 8 weeks leading to euthanasia within 2 weeks. However, this is in contrast to the study conducted by Zwick et al. (27) in which four rhesus macaques were intracerebrally inoculated with BoDV-1, of which only one animal became symptomatic succumbing to encephalitis on 67 dpi.

As mentioned before, there has been no previous VSBV-1 NHP study. While neurological signs were prominent in animals intracerebrally infected with BoDV-1 (group 3), they were less severe in the intracerebrally inoculated VSBV-1 animals (group 1) that rather showed behavioral changes. The onset of clinical disease with the VSBV-1-infected animals was delayed by about 1 to 2 weeks and disease duration prolonged compared to the BoDV-1-infected animals.

Only one (NHP #12) of six animals was successfully infected with a bornavirus by the peripheral routes. In this case, it took 3 weeks longer than for the BoDV-1 intracerebrally inoculated animals until first neurological signs became apparent. However, disease progression in this animal was similar to the intracerebrally inoculated animals (Figure 1 and Figure S1, Supplementary Material). In the remaining five animals, no viral RNA or antigen could be detected in any organ investigated at the end of the study. However, NHP #11 showed transient mild neurologic signs following BoDV-1 infection by the peripheral routes. Additionally, one CSF sample from this animal was weakly BoDV-1 RNA-positive. Our data indicate that, peripheral route exposure seems unlikely to be highly effective but also demonstrates the possibility to establish an infection. Therefore, future studies need to define the most effective peripheral route as well as the dose of inoculation to address an important public health question.

Neither the entry site of infection nor the incubation period is known for any human infections with mammalian bornaviruses. The only exception is the iatrogenic BoDV-1 organ transplant cluster (17). The three recipients showed first clinical symptoms within 3 to 4 months following transplantation and two of them died 6 to 7 months thereafter. It should be noted, however, that all three humans were highly immunocompromised (17). For horses it is hypothesized that BoDV-1 infection might occur through exposure to excretions from infected shrews through intranasal uptake (29). Whether this route is also responsible for human infection is unknown and should be addressed in future NHP studies. So far, all other reported human BoDV-1 cases have a more rapid disease progression with deaths occurring within 2 to 8 weeks after hospitalization. Symptoms included fever, headache, confusion, psychomotor slowing, myoclonus, unsteady gait, deep coma, and deteriorating brain stem reflexes subsequently leading to death (18–21). VSBV-1 infection in humans slightly differs with clinical symptoms including shivers, fever, confusion, mental disorders, reduced movement, gait disturbances, muscle twitching, and finally coma. These patients succumb to infection between 2 and 4 weeks after disease onset (1, 4). For VSBV-1 no data on transmission mode exists so far. Although, a subcutaneous and/or intramuscular infection route is suggested given that one infected squirrel breeder reported sustaining scratches and bites from their variegated squirrels (1).

Viral shedding was not observed or investigated in human mammalian bornavirus cases nor in the two previous BoDV-1 rhesus macaque studies (26, 27). We, therefore, analyzed oral, nasal, and rectal swabs on a weekly basis, but virus detection was limited to a few BoDV-1 genome positive oral swabs shortly after onset of disease. Urine was only positive for one BoDV-1-infected animal (NHP #12; Table 2A). Virus isolation was negative on all RNA positive swabs and urine samples. Therefore, viral shedding seems to only sporadically occur and, therefore, seems unlikely to cause human-to-human transmission.

For VSVB-1 and BoDV1, viral RNA distribution in tissues showed clear neurotropism with viral genome copies in the brain of up to 1.24 × 10^10^ per mg. For VSBV-1, RT-qPCR detected viral RNA exclusively in the central nervous system, whereas for BoDV-1 a few additional organs were positive. Further analysis by IHC revealed that in those organs only peripheral nerves contained viral antigen (Figure 2 and Figure S2, Supplementary Material) similar to what has been described from BoDV-1-infected horses. Viral RNA detection in salivary gland and bladder explain RNA-positive oral swabs and urine samples. All four BoDV-1-infected NHPs showed RT-qPCR-positive CSF results at necropsy. Unfortunately, VSBV-1 could only be detected in CSF samples from two of the three intracerebrally infected NHPs. Thus, CSF may not be reliable as a clinical specimen for the diagnosis of mammalian bornavirus infections. A lack of BoDV-1 RNA in CSF samples has been previously reported from human cases (21) confirming the suitability of the NHP for bornavirus infection.

Virus-specific antibodies could be detected in sera from all three intracerebrally infected VSBV-1 animals as well as in one CSF sample. For BoDV-1, two of the four animals with detectable virus (one intracerebral and one peripheral) seroconverted (Table 2B). BoDV-1 antibodies were reported in a previous rhesus macaque study with titers up to 1:16 (26). This, however, is below the chosen cut-off of our assay and we, therefore, may have missed low-titered antibody responses. In contrast, almost all infected patients developed high serum antibody titers to bornavirus infection with low-titered CSF antibodies later in infection. At the time of hospital admission, antibody titers were sometimes low or undetectable (17, 18, 21). The early endpoint in our NHP study might be one reason for this difference, especially for BoDV-1 infection which resulted in a faster disease progression with insufficient time to prime the immune system.

The most significant histological findings were severe lymphocytic perivascular cuffing and meningeal inflammatory infiltrates, which were more obvious in the intracerebrally infected animals and NHP #12, the only animal that succumbed to BoDV-1 infection through peripheral route inoculation (Table S2, Supplementary Material). Interestingly, Wallerian degeneration of the spinal cord was identified in all peripherally infected animals but only one intracerebrally infected animal (NHP #1). Wallerian degeneration occurs when an axon is severed or injured and develops retrograde degeneration with axonal swelling, infiltration of Gitter cells, and loss of myelin. Focal areas of Wallerian degeneration would be expected in the brains and spinal cords of animals who were subjected to cerebral biopsies and spinal fluid collections. Therefore, it is likely that the degenerative lesions in this study are iatrogenic, which is supported by the appearance in the peripherally infected animals that were subject to more procedures due to prolonged study progression. In all other animal models, Wallerian degeneration was also not found further supporting the iatrogenic origin. Interestingly, retinopathy as described by Stitz et al. (26) was not found in our study animals.

In conclusions, all VSBV-1 and BoDV-1 intracerebrally as well as one peripherally BoDV-1-infected animals developed lethal meningoencephalitis similar to what has been observed in human cases (1, 4, 17–21). As such, bornavirus infection in rhesus macaques recapitulates human-like disease and, thus reflects a surrogate model for accidental dead-end host infection. The established NHP disease model will be instrumental for further pathogenesis studies and countermeasure development.

## Materials and Methods

### Ethics and biosafety

All animal studies were approved by the Animal Care and Use Committee of the Rocky Mountain Laboratories, NIH and conducted in an Association for Assessment and Accreditation of Laboratory Animal Care (AAALAC) International accredited facility. Work was carried out according to the institutional guidelines for animal use and followed the guidelines and basic principles in the United States Public Health Service Policy on Humane Care and Use of Laboratory Animals, the Animal Welfare Act and the Guide for the Care and Use of Laboratory Animals. The Institutional Biosafety Committee (IBC) approved work with bornaviruses in BSL-3 biocontainment. Sample inactivation was performed according to IBC-approved standard operating procedures for removal of specimens from high biocontainment.

Rhesus macaques were housed in a climate-controlled room with a fixed light–dark cycle (12-hours light/12-hours dark) in adjacent, individual primate cages allowing social interactions. Teklad Global 25% Protein Primate Diet 2055, (Teklad Global Diets®, Madison, WI) was provided twice per day and supplemented with treats, fruits, and vegetables. Water was available ad libitum. Environmental enrichment consisted of a variety of human interactions, commercial toys, videos, and music. Animals were monitored at least twice per day throughout the study.

### Animal study design

A total of 12 rhesus macaques (*Macaca mulatta*, seven males, five females; 18 to 24 months old and body weight of approximately 3 kg) were infected with either VSBV-1 (six animals) or BoDV-1 (six animals). In each group, three animals were infected by intracerebral (i.c.) inoculation and three animals were infected by a combination of peripheral routes (intranasal, intraocular, subcutaneous, and intramuscular; Table 1). In each case, a virus-infected rat brain suspension was used (approximately 2 × 10^4^ focus forming units (FFU)). Prior to infection, a physical exam was conducted and swabs (oral, nasal, and rectal), blood, and cerebrospinal fluid (CSF) were collected. On the day of infection, weekly postinfection and at necropsy, a physical exam was conducted and swabs (oral, nasal, and rectal) and blood were collected. CSF was collected monthly postinfection and at necropsy. Samples were used for haematological, serological, immunological, and virologic analyses. Animals were monitored and scored daily using an established scoring sheet (30). Daily scoring included assessment of general appearance, respiratory effort/pattern, urine/feces output, appetite, and activity level. Numerical values were assigned to the assessment parameters and a combined score of ≥ 35 indicated the animal met euthanasia criteria. Animals that did not meet euthanasia criteria were euthanized 20 weeks postinfection as end point of this study. If animals did not show disease or had only mild clinical signs 11 weeks after infection (day 77/78 postinfection), brain biopsies were performed (Table 1). All animals were necropsied and the following tissues were collected: injection site(s), lung, heart, liver, spleen, kidney, urinary bladder, urine, salivary gland, cervical lymph node, brachial plexus, sacral plexus, cervical spinal cord, and optic nerve. One brain hemisphere en bloc was placed in formalin for histology. The other hemisphere was dissected into olfactory bulb, frontal lobe, parietal lobe, occipital lobe, temporal lobe, cerebellum, pons, medulla oblongata, thalamus, hypothalamus, and hippocampus and placed in formalin. Tissue samples were used for virological, immunological, and pathological analyses.

### BoDV-1 and VSBV-1 inoculum

BoDV-1 strain “H24” (31) and VSBV-1 strain “Theodore” (25) from infected Lewis rat brains were used for inoculum preparation. Rat brains were homogenized in 2 ml MEM (H) + MEM (E) media and sonicated (Brandson, Sonifier 450, Emerson, St Louis, MO, USA). Supernatant was clarified from debris through centrifugation and titrated on Vero 76 cells (Collection of Cell Lines in Veterinary Medicine CCLV-RIE 228). Both inocula were screened for contaminations by high-throughput sequencing and subsequent analysis of the raw reads using the pipeline RIEMS (32). Within the VSBV-1 preparation, two reads of a retroviral rat virus were detected. No further contaminants were discovered. In addition, the bornaviral whole genome sequences were assembled using the “VirBaits” set (33) containing 3,279 bornavirus-specific RNA baits for enrichment of viral nucleic acids. Virus stocks were adjusted to contain approximately 2 × 10^4^ FFU in 200 µl or 400 µl for intracerebral and peripheral routes of infection, respectively. Viral titres were confirmed by titration on Vero 76 cells.

### Virus titration

Virus titrations were performed by endpoint titration in Vero 76 cells. Cells were inoculated with 10-fold serial dilutions of swab samples, CSF, urine, or tissue homogenates in quadruplicates. Cells were fixed after 5 days using paraformaldehyde and triton X, and incubated with a 1:500 diluted rabbit anti-BoDV-1-P (34) antibody for 45 min. Afterwards, cells were stained with an FITC-labeled goat-antirabbit IgG antibody (Sigma-Aldrich, St. Louis, MO, USA). TCID_50_ was adjusted for tissue weight and calculated by the method of Spearman–Karber. The limit of detection for the titration assay is 101.625 TCID50.

### Peripheral inoculation: combined intranasal, conjunctival, intramuscular, and subcutaneous injection

Animals were sedated by intramuscular injection of 10 mg/kg ketamine (Zetamine™, VetOne/MWI, Boise, ID) and inoculated via the following routes: ocular (25 μl/eye), intranasal (50 μl/naris), intramuscular (100 μl in the left caudal thigh), and subcutaneous (150 μl equally divided over three sites in the subscapular region).

### Intracerebral inoculation

Animals were anesthetized with an intramuscular injection of 3 mg/kg Telazol™ (Zoetis, Kalamazoo, MI), intubated, maintained on isoflurane (Fluriso™, VetOne/MWI, Boise, ID), and connected to monitoring equipment. Procedural monitoring included electrocardiography, pulse oximetry, capnography, noninvasive blood pressure, and body temperature (SurgiVet Advisor®, Smiths Medical, Dublin, OH). Heat support was provided using a forced air warming unit (3M Bair Hugger™, Maplewood, MN). Buprenorphine HCl (Par Pharmaceutical, Spring Valley, NY) was administered preoperatively (0.03 mg/kg i.m.). The hair on the dorsal head was clipped and the head secured in a stereotaxic device. The surgical site was prepped with DuraPrep™ (3M,  St. Paul, MN), allowed to dry and draped for surgery. An approximately 3-cm incision was made over the right frontal lobe using a #15 scalpel blade. Blunt dissection was utilized to retract the muscles and allow visualization of the skull. A Barraquer eye speculum was placed to aid in visualization. The periosteum was incised and retracted laterally using a #15 scalpel blade. Trepanation was performed using a 1.85-mm round tip burr and drill (Micro-Drill Kit, Braintree Scientific, Braintree, MA). Once the dura mater was exposed, 200 µl of inoculum was injected into the frontal lobe, approximately 5-mm deep with a 25G needle. A piece of bone wax (Medline Industries, Mundelein, IL) was utilized to seal the burr hole in the skull. The muscles were repositioned over the site and the skin edges were closed with surgical skin staples (Medline) and Dermabond® tissue adhesive (Ethicon™, Somerville, NJ). Meloxicam (OstiLox™, VetOne/MWI, Boise, ID) was administered immediately postoperatively (0.2 mg/kg s.c.) and then once daily for 3 days (0.1 mg/kg s.c.). The procedure was performed by American College of Laboratory Animal Medicine board certified veterinarians.

### Brain biopsy

For brain biopsies, the animals were anesthetized and monitored as described for the intracerebral inoculation. This time, Buprenorphine HCl SR (ZooPharm, Laramie, WY) was administered preoperatively (0.2 mg/kg s.c.). The surgical preparation and approach were performed as described above. After the trepanation, four small brain samples were collected from the frontal lobe utilizing an Acu-Dispo-Curette® 1 mm dermal cup curette (Acuderm Inc., Ft. Lauderdale, FL). A piece of bone wax (Medline) was utilized to seal the burr hole in the skull. The muscles were repositioned over the site and the skin edges were closed with surgical skin staples (Medline) and Dermabond® tissue adhesive (Ethicon™, Somerville NJ). The procedure was performed by American College of Laboratory Animal Medicine board certified veterinarians.

### CSF collection

Animals were anesthetized with an intramuscular injection of 3 mg/kg Telazol™ (Zoetis) and supplemented with isoflurane (Fluriso™, VetOne/MWI) via face mask as needed. The hair on the dorsal neck and head was clipped and the site was prepped with DuraPrep™ (3M) and allowed to dry. The head was positioned and held in complete ventroflexion. A 22G x 1.5” Quincke spinal needle (Becton Dickinson, Franklin Lakes, NJ) was inserted into the suboccipital space and advanced until the flow of CSF was observed. Once CSF flow was observed, a 1-cc syringe was attached, and 0.2 to 0.5 ml of CSF was collected. Following collection, the needle was removed, and direct pressure was applied to the puncture site. The procedure was performed by American College of Laboratory Animal Medicine board certified veterinarians.

### Hematology and clinical chemistry

Hematological analysis was completed on a ProCyte Dx® (IDEXX Laboratories, Westbrook, ME) and the following parameters were evaluated: red blood cells (RBC); hemoglobin (Hb); hematocrit (HCT); mean corpuscular volume (MCV); mean corpuscular hemoglobin (MCH); mean corpuscular hemoglobin concentration (MCHC); red cell distribution weight (RDW); platelets; mean platelet volume (MPV); white blood cells (WBC); neutrophil count (absolute and percentage); lymphocyte count (absolute and percentage); monocyte count (absolute and percentage); eosinophil count (absolute and percentage); and basophil count (absolute and percentage). Serum chemistry analysis was completed on a VetScan VS2® Chemistry Analyzer (Abaxis, Union City, CA) and the following parameters were evaluated: glucose; blood urea nitrogen (BUN); creatinine; calcium; albumin; total protein; alanine aminotransferase (ALT); aspartate aminotransferase (AST); alkaline phosphatase (ALP); total bilirubin; globulin; sodium; potassium; chloride, and total carbon dioxide.

### Pathology: necropsy, histopathology, and IHC

Tissues were fixed in 10% neutral buffered formalin for a minimum of 7 days including two changes of formalin. Tissues were placed in cassettes and processed using a Sakura VIP-6 Tissue Tek tissue processor (Sakura, Torrance, CA, USA) on a 12-hour automated schedule, using a graded series of ethanol, xylene, and PureAffin and embedded in Ultraffin paraffin polymer (Cancer Diagnostics, Durham, NC, USA). Samples were sectioned at 5 µm, and resulting slides were dried overnight at 42°C. Slides were stained with hematoxylin and eosin for histopathology. For IHC, tissues were stained with a 1:500 diluted BoDV-1 P rabbit polyclonal antibody (34), with a high cross reactivity for VSBV-1. The antibody was detected using ImmPress-VR antirabbit IgG polymer kit (Vector Laboratories, MP-6401–15, no dilution, Burlingame, CA, USA) followed by Discovery purple kit (Roche Tissue Diagnostics, Tucson, AZ, USA). For dual stain IHC, three additional antibodies were used: Iba-1 (for macrophage and microglia detection; in-house antibody, polyclonal 1:2,000 diluted), GFAP (for astrocyte detection; 1:1,000 diluted, monoclonal, Novus Biologicals, Littleton, CO, USA) and NFP (for neuron detection; 1:250 diluted, polyclonal, Agilent, Santa Clara, CA, USA). These antibodies were detected by the following secondary antibodies: ImmPRESS-VR antirabbit IgG polymer kit, ImmPRESS-AP antimouse IgG polymer kit, and ImmPRESS-AP antirabbit IgG polymer kit (Vector Laboratories, MP-6401–15, no dilution). Discovery was enabled by the Discovery purple and yellow kit (Roche Tissue Diagnostics).

### RNA extraction, detection, and dideoxy chain-termination sequencing of bornaviruses

RNA was extracted from swabs, blood, and CSF using the QIAmp Viral RNA kit (Qiagen, Hilden, Germany) according to the manufacturer's instructions. Tissues (30 mg) were homogenized in RLT buffer and RNA was extracted using the RNeasy kit (Qiagen) according to the manufacturer's instructions. RNA was eluted in 50 µl total volume. Bornaviral RNA was detected by RT–qPCR using a VSBV-1 specific (Squirrel mix 10.1) (25) or a BoDV-1 specific assay (BoDV-1 mix 1) (17) and the qScript XLT one-step RT-qPCR ToughMix Kit (Quanta BioSciences, Gaithersburg, MD, USA). In each run, standard dilutions of quantified RNA standards were run in parallel, to calculate copy numbers in the samples. All RT-qPCRs were performed on a Rotorgene Q (Rotorgene Q software version 2.3.1; Qiagen). Dideoxy chain-termination sequencing for VSBV-1 and sequence analysis was done as described elsewhere (3, 25). For BoDV-1, primers are given in Table S1 (Supplementary Material).

### Detection of bornavirus-reactive serum antibodies

Sera were screened for the presence of bornavirus-reactive IgG by indirect immunofluorescence assays (iIFA). Briefly, BoDV-1 He80-infected cells were mixed 1:5 with uninfected Vero 76 cells and incubated overnight at 37°C and 5% CO_2_. Cells were fixed and permeabilized, then, incubated 45 min with rhesus macaque sera serially diluted two-fold (starting from 1:20). For visualization, cells were incubated with a 1:300 diluted Alexa Fluor 555-labeled mouse–antimonkey–IgG antibody (Southern Biotech, Birmingham, AL, USA) for another 45 min. Cells were analyzed by fluorescent microscopy in a 96-well microtiter plate, as previously described (14,18).

### T-cell assay

PBMCs were prepared by centrifugation on a Ficoll gradient. Cells (3 × 10^6^ PBMCs) from each rhesus monkey were seeded into duplicate wells in a 96-well flat-bottom plate precoated with a human IFN-γ and TNF-α capturing antibody Human IFN-γ/TNF-α Double-Color ELISPOT (ImmunoSpot, Bonn, Germany). PBMCs were stimulated with BoDV-1 or VSBV-1 at a MOI of 1, 0.1, and 0.01, for 24 hours in a humidified incubator with 5% CO_2_ at 37°C. IFN-γ and TNF-α spots were developed according to the manufacturer's protocol.

## Funding

This work was supported by grants from the Federal Ministry of Education and Research: project ZooBoCo (Zoonotic Bornavirus Consortium), within the research network for zoonotic infectious diseases, grant no. 01KI1722A/01KI2005A and by the Intramural Research Program, National Institutes of Allergy and Infectious Diseases (NIAID), National Institutes of Health (NIH).

## Supplementary Material

pgac073_Supplemental_File

## Data Availability

All data is included in the manuscript and/or supporting information.
