# Supplementary material for: Development of a nonhuman primate model for mammalian bornavirus infection
Source: PNAS Nexus. 2022 Jun 8;1(3):pgac073. doi: 10.1093/pnasnexus/pgac073 (PMC9291224; doi:10.1093/pnasnexus/pgac073)
Supplement: pgac073_Supplemental_File [file pgac073_supplemental_file.docx]

# Supplementary material

# Supplementary tables

Table S1: Primers used for dideoxy chain termination sequencing of Borna disease virus 1.

| **Name** | **Sequence 5´- 3´** | **Expected amplicon Length** |
| --- | --- | --- |
| BoDV-1-F | ATG CCA CCC AAG AGA CGC | 1021 bp |
| BoDV-1021-R | GCC TAT ACC GGG CAA GCT G |  |
| BoDV-300-F | CCT GTC GAC GCC TGT TAC | 1000 bp |
| BoDV-1300-R | ACC CCG ATC GTT CCC GTC |  |
| BoDV-605-F | GGG TAG GCT CCT TTG TGT TGT | 995 bp |
| BoDV-1600-R | AGT GGT CGC ACC GCT GAG |  |
| BoDV-913-F | GCA GCG TTT TAC TGG AGT AAG A | 987 bp |
| BoDV-1900-R | TGG GCC ATC CAG GGA CGA T |  |
| BoDV-1204-F | TAG TCA GGA GGC TCA ATG GC | 924 bp |
| BoDV-2128-R | TGA TTG CGT GCT TGC TCT G |  |
| BoDV-1500-F | GGG CAC TCT TGG AGA CAT CT | 1000 bp |
| BoDV-2500-R | TTA ACA GTA CCA GTG TAC CGA TTG |  |
| BoDV-1827-F | AAA TCG AAT CAC CAT GAA TTC AAA ACA | 948 bp |
| BoDV-2775-R | GTC CCA TTG AGG GTT TTG TTC A |  |
| BoDV-2113-F | GTG TAC AAG GAC CCG ATT AG | 1037 bp |
| BoDV-3150-R | GTG ACA TTA ACA CGA TGG CAT T |  |
| BoDV-2405-F | TCA TGC CTC AAG TAC CAC TG | 981 bp |
| BoDV-3386-R | TTG CCT CAA ACT CCT CCA G |  |
| BoDV-2693-F | GCA ATG CTG AGC GAT GGC A | 1014 bp |
| BoDV-3707-R | TGG TCG GTA CGG TTT ATT CCT |  |
| BoDV-3000-F | AGA TCC TCC AGT CTC TGC T | 1000 bp |
| BoDV-4000-R | TCA AGT TGG GCT CGG ACC |  |
| BoDV-3334-F | GAT TCG GTA CAA TAA GAC CTC A | 976 bp |
| BoDV-4310-R | TGG GGG AGG TTG ATT GAG T |  |
| BoDV-3643-F | CTT ACC AGC CTC CTT CGC | 953 bp |
| BoDV-4596-R | GGC TTG GGG CGT TTC CAG |  |
| BoDV-3900-F | GGC TAT ACT AAC AGT TAT AGT ATC | 1031 bp |
| BoDV-4913-R | TAT TTC TTC CAA AAG GCC GCT |  |
| BoDV-4200-F | ATG CCA TCT CCT AAT CGC CT | 1001 bp |
| BoDV-5201-R | TCA TGG TCA TTG AGT GTG TCT TTA |  |
| BoDV-4500-F | GGT GCA GAA GTC ATG GTA CTT | 966 bp |
| BoDV-5466-R | GTC TTC AAC GGG CTC ACC |  |
| BoDV-4800-F | TGC TGA CCT AGA TAT GGA TC | 1081 bp |
| BoDV-5818-R | ACC CGC GAG AGC TGC TT |  |
| BoDV-5103-F | CTC GAA GCA AAC ATT GGC AAT C | 999 bp |
| BoDV-6102-R | AAA GGG CAG TGG GTC GGA |  |
| BoDV-5378-F | CTG GGT GCA CAC TGC CAT | 1022 bp |
| BoDV-6400-R | GGG GTC GAA GAG GCA TCA T |  |
| BoDV-5700-F | TGG CCA TAA CCT CAA GGT AGA | 1000 bp |
| BoDV-6700R | GGA ATT GCT CGG CAA CAA AC |  |
| BoDV-6006-F | GAC CCT CTG CCT CGT AGG | 999 bp |
| BoDV-7005-R | AGC GTC AGA TGT GTA CCC C |  |
| BoDV-6300-F | CTC ACA CTC GTC ACG CAT A | 1000 bp |
| BoDV-7300-R | CGA ACT CCT CTG AGT CTA TCT T |  |
| BoDV-6600-F | TTT GGA AGG TTG CAC TTA CCT T | 1010 bp |
| BoDV-7610-R | TTC TCA AAC TGA AGG AGC CG |  |
| BoDV-6937-F | GTT ACC GAT CCA CAC TTG AAC | 974 bp |
| BoDV-7911-R | AAA CTG TGT GGG CTT CAC G |  |
| BoDV-7200-F | TGC TGA TTC GGT GAT TAG ATC G | 981 bp |
| BoDV-8181-R | ACT TGA CAC AGC CCC GCA |  |
| BoDV-7541-F | TTC CGT GGA GTA TTG TAA TTC G | 947 bp |
| BoDV-8488-R | CTC GGA TCA TAA TGA GGT CCA T |  |
| BoDV-7800-F | AGT GGA GAT TGA AGT GTC TGG | 1018 bp |
| BoDV-8818-R | CAC CAC TGA CAT ATG GTT TTT TAA |  |
| BoDV-7989-F | TCA ATC ACA GGT CCT AAA GAT G | 781 bp |
| BoDV-8770-R | CTY AAT CAA YYA HCA ATA ACT GCG |  |
| BoDV-1-8750-F | GTG ATG AGG ATT GTC AGG G | 565 bp |
| BoDV-1-401-R | GCA GTC TTA ACA ACA GTC TG |  |
| BoDV-1-8632-F | TGT YTA CTA CGT TGG RGT TGT | 516 bp |
| BoDV-1-234-R | GCC ACT GCG TTC TGC CT |  |

Table S2: Histopathology results of the necropsied rhesus macaques. Histologic sections of the specified organs were scored from 0 to 5 based on the following percent and severity of affected pathology.

|  | #1 | #2 | #3 | #4 | #5 | #6 | #7 | #8 | #9 | #10 | #11 | #12 |
| --- | --- | --- | --- | --- | --- | --- | --- | --- | --- | --- | --- | --- |
| Virus / route | VSBV-1 i.c. | | | VSBV-1 non-i.c. | | | BoDV-1 i.c. | | | BoDV-1 non-i.c. | | |
| euthanized | d56 | d71 | d84 | d140 | d140 | d140 | d41 | d40 | d35 | d140 | d140 | d59 |
| brain biopsy | N | N | Y | Y | Y | Y | N | N | N | Y | Y | N |
| Skin |  |  |  |  |  |  |  |  |  |  |  |  |
| dermatitis, lymphocytic | 0 | 0 | 1 | 0 | 0 | 0 | 0 | 0 | 0 | 0 | 0 | 0 |
| Brachial Plexus |  |  |  |  |  |  |  |  |  |  |  |  |
| lyphocytic infiltrate | 0 | 0 | 0 | 0 | 0 | 0 | 1 | 2 | 0 | 0 | 0 | 1 |
| Cervical LN |  |  |  |  |  |  |  |  |  |  |  |  |
| draininig neutrophils with eosinophils | 0 | 1 | 0 | 0 | 0 | 0 | 0 | 0 | 0 | 0 | 0 | 0 |
| draining erythrocytes | 0 | 0 | 1 | 0 | 0 | 0 | 0 | 0 | 0 | 0 | 0 | 0 |
| Salivary Gland |  |  |  |  |  |  |  |  |  |  |  |  |
| lymphocytic infiltrate, periductular | 2 | 0 | 0 | 0 | 2 | 2 | 0 | 2 | 0 | 1 | 1 | 1 |
| Muscle |  |  |  |  |  |  |  |  |  |  |  |  |
| lymphocyic infiltrate with myocyte degeneration | 0 | 0 | 0 | 0 | 0 | 0 | 0 | 0 | 0 | 0 | 0 | 2 |
| Heart |  |  |  |  |  |  |  |  |  |  |  |  |
| myocardial infiltrate - lymphocytic | 0 | 0 | 1 | 1 | 1 | 1 | 0 | 1 | 0 | 0 | 0 | 0 |
| Liver |  |  |  |  |  |  |  |  |  |  |  |  |
| lymphocytic infiltrate periportal and random | 0 | 0 | 0 | 0 | 0 | 1 | 0 | 1 | 0 | 1 | 1 | 0 |
| vacuolar change - glycogen type | 0 | 3 | 0 | 0 | 0 | 0 | 0 | 0 | 0 | 0 | 0 | 0 |
| Spleen |  |  |  |  |  |  |  |  |  |  |  |  |
|  | 0 | 0 | 0 | 0 | 0 | 0 | 0 | 0 | 0 | 0 | 0 | 0 |
| Kidney |  |  |  |  |  |  |  |  |  |  |  |  |
| infiltrate, interstitial, lymphocytic | 1 | 1 | 1 | 1 | 0 | 1 | 0 | 1 | 0 | 1 | 0 | 1 |
| Urinary |  |  |  |  |  |  |  |  |  |  |  |  |
| perivascular lymphocytic infiltrate, lamina propria | 0 | 0 | 1 | 0 | 0 | 0 | 0 | 0 | 0 | 1 | 0 | 1 |
| Cervical Spinal Cord |  |  |  |  |  |  |  |  |  |  |  |  |
| wallerian degeneration | 1 | 0 | 0 | 2 | 2 | 1 | 0 | 0 | 0 | 1 | 2 | 3 |
| spheroids | 0 | 0 | 0 | 0 | 2 | 1 | 0 | 0 | 0 | 0 | 0 | 0 |
| gliosis, gray matter | 1 | 1 | 0 | 0 | 0 | 0 | 0 | 0 | 0 | 0 | 0 | 0 |
| Sacral Spinal Cord |  |  |  |  |  |  |  |  |  |  |  |  |
| lymphocytic infiltrate | 0 | 0 | 0 | 0 | 0 | 0 | 0 | 1 | 0 | 0 | 0 | 0 |
| Optic Nerve |  |  |  |  |  |  |  |  |  |  |  |  |
| lymphocytic and eosinophilic infiltrate - perivascular | 2 | 0 | 3 | 0 | 0 | 0 | 0 | 0 | 0 | 0 | 0 | 0 |
| Lung |  |  |  |  |  |  |  |  |  |  |  |  |
| BALT: lymphocytic nodules, perivascular | 2 | 1 | 0 | 3 | 1 | 2 | 2 | 2 | 0 | 2 | 1 | 2 |
| exudate, mucinous, bronchiolar | 2 | 0 | 0 | 0 | 0 | 0 | 0 | 0 | 0 | 0 | 0 | 0 |
| Edema | 2 | 0 | 0 | 0 | 0 | 0 | 0 | 0 | 0 | 0 | 0 | 0 |
| interstitial infiltrates with alveolar macrocytosis | 0 | 0 | 0 | 0 | 0 | 0 | 0 | 1 | 0 | 0 | 0 | 0 |
| Eye |  |  |  |  |  |  |  |  |  |  |  |  |
|  | 0 | 0 | 0 | 0 | 0 | 0 | 0 | 0 | n/p | 0 | 0 | 0 |
| Cerebrum |  |  |  |  |  |  |  |  |  |  |  |  |
| leucomalacia with abundant gitter cells with intracytoplasmic brown globular pigment | 3 | 0 | 3 | 1 | 0 | 3 | 3 | 3 | 0 | 0 | 2 | 0 |
| lymphocytic perivascular cuffing with eosinophils | 4 | 4 | 4 | 0 | 0 | 1 | 1 | 1 | 4 | 0 | 0 | 2 |
| cortex, subacute nodular lymphohistiocytic and eosinophilic | 0 | 0 | 0 | 0 | 0 | 0 | 0 | 0 | 0 | 1 | 1 | 0 |
| mineralization | 0 | 0 | 0 | 0 | 0 | 0 | 0 | 0 | 0 | 0 | 1 | 0 |
| meningeal infiltrate - lymphocytic | 3 | 3 | 1 | 0 | 0 | 0 | 0 | 1 | 3 | 0 | 0 | 1 |
| Cerebellum |  |  |  |  |  |  |  |  |  |  |  |  |
| lymphocytic perivascular cuffing with eosinophils | 0 | 0 | 0 | 0 | 0 | 0 | 0 | 0 | 0 | 0 | 0 | 0 |
| Brainstem |  |  |  |  |  |  |  |  |  |  |  |  |
| lymphocytic perivascular cuffing | 2 | 3 | 2 | 0 | 0 | 0 | 0 | 3 | 1 | 0 | 0 | 0 |
| wallerian degeneration | 3 | 0 | 0 | 0 | 0 | 0 | 0 | 4 | 0 | 0 | 0 | 0 |
| spheroids | 2 | 0 | 0 | 0 | 0 | 0 | 0 | 0 | 0 | 0 | 0 | 0 |
| abundant gitter cells with intracytoplasmic brown globular pigment | 3 | 0 | 0 | 0 | 0 | 0 | 0 | 4 | 0 | 0 | 0 | 0 |

Histologic sections of the specified organs were scored from 0 to 5 based on the following percent and severity of affected pathology. 0 = no lesion, 1 = minimal (1-10%), 2 = mild (11-25%), 3 = moderate (26-50%), 4 = marked (51-75%), 5 = severe (76-100%); percentage = number of cells per affected area. n.p. = not present. i.c. = intracerebral; non-i.c. = peripheral routes

Table S3: Immunohistochemistry results of selected tissues from the bornavirus inoculated rhesus macaques.

|  | #1 | #2 | #3 | #4 | #5 | #6 | #7 | #8 | #9 | #10 | #11 | #12 |
| --- | --- | --- | --- | --- | --- | --- | --- | --- | --- | --- | --- | --- |
| Virus / route | VSBV-1 i.c. | | | VSBV-1 non-i.c. | | | BoDV-1 i.c. | | | BoDV-1 non-i.c. | | |
| euthanized | d56 | d71 | d84 | d140 | d140 | d140 | d41 | d40 | d35 | d140 | d140 | d59 |
|  |  |  |  |  |  |  |  |  |  |  |  |  |
| Brain–IHC immunoreactivity | 4 | 4 | 2 | 0 | 0 | 0 | 5 | 5 | 5 | 0 | 0 | 5 |
| Brain |  |  |  |  |  |  |  |  |  |  |  |  |
| neurons, astrocytes | 4 | 4 | 3 | 0 | 0 | 0 | 5 | 5 | 5 | 0 | 0 | 5 |
| Spinal cord, cervical |  |  |  |  |  |  |  |  |  |  |  |  |
| neurons | 3 | 4 | 0 | 0 | 0 | 0 | 5 | 5 | 5 | 0 | 0 | 5 |
| Lung |  |  |  |  |  |  |  |  |  |  |  |  |
|  |  |  |  |  |  |  |  |  | 0 |  |  |  |
| Brachial plexus |  |  |  |  |  |  |  |  |  |  |  |  |
|  |  |  |  |  |  |  | 5 | 5 | 5 |  |  | 5 |
| Skin |  |  |  |  |  |  |  |  |  |  |  |  |
| nerve |  |  |  |  |  |  | 4 | 4 | 1 |  |  | 2 |
| Cervical lymphe nodes |  |  |  |  |  |  |  |  |  |  |  |  |
| subcutaneous nerves in adipose |  |  |  |  |  |  | 2 | 5 | 2 |  |  | 3 |
| Salivary gland |  |  |  |  |  |  |  |  |  |  |  |  |
| subcutaneous nerves in adipose |  |  |  |  |  |  | 1 | 3 | 0 |  |  | 0 |
| urinary bladder |  |  |  |  |  |  |  |  |  |  |  |  |
| nerve |  |  |  |  |  |  |  |  |  |  |  | 2 |

0 = none, 1 = rare/few cells, 2 = scattered, 3 = moderate, 4 = numerous, 5 = diffuse; : i.c. = intracerebral; non-i.c. = peripheral routes

# Supplementary figures


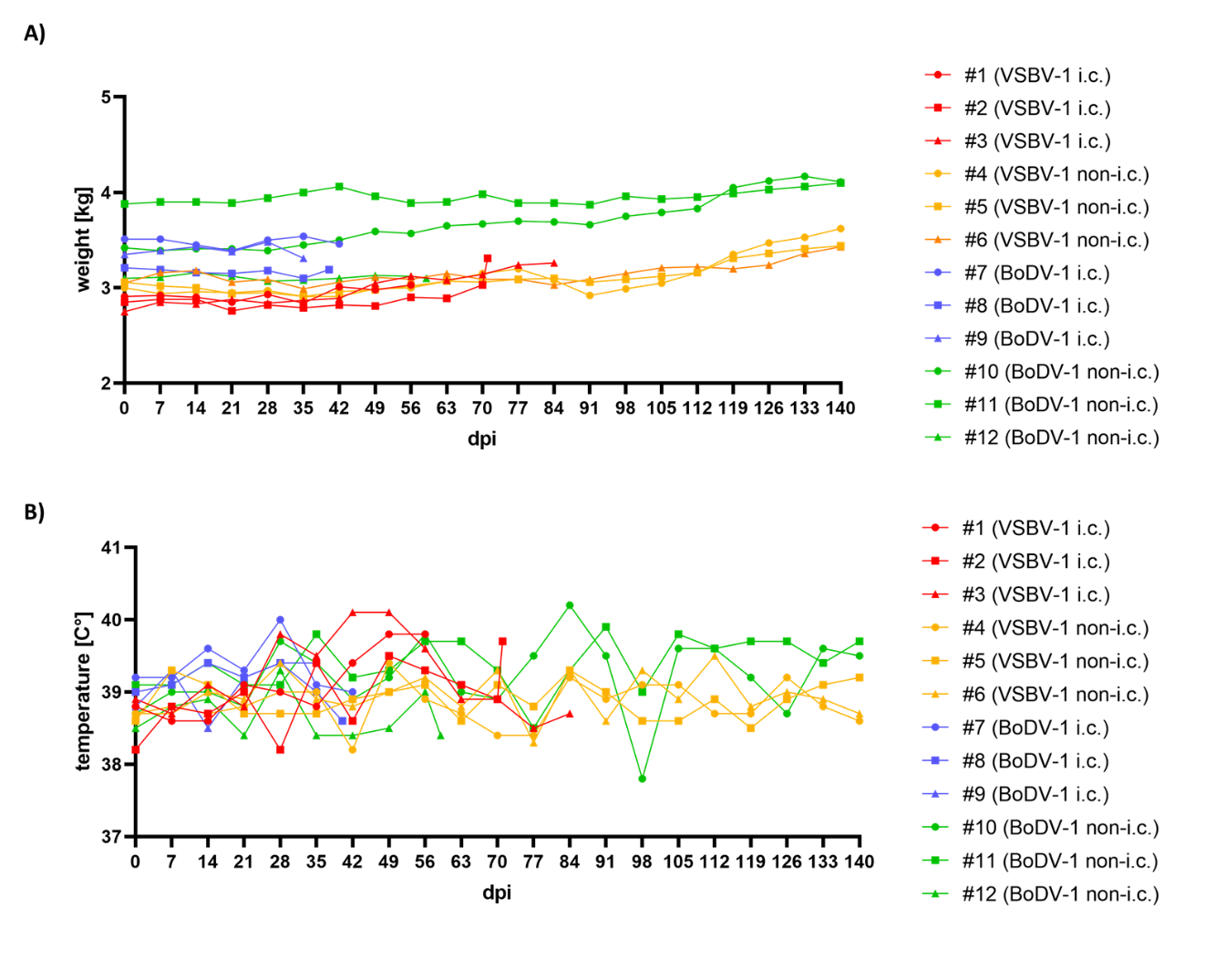


Figure S1: Weight and temperature of the twelve rhesus macaques throughout the study. i.c. = intracerebral; non-i.c. = peripheral routes.


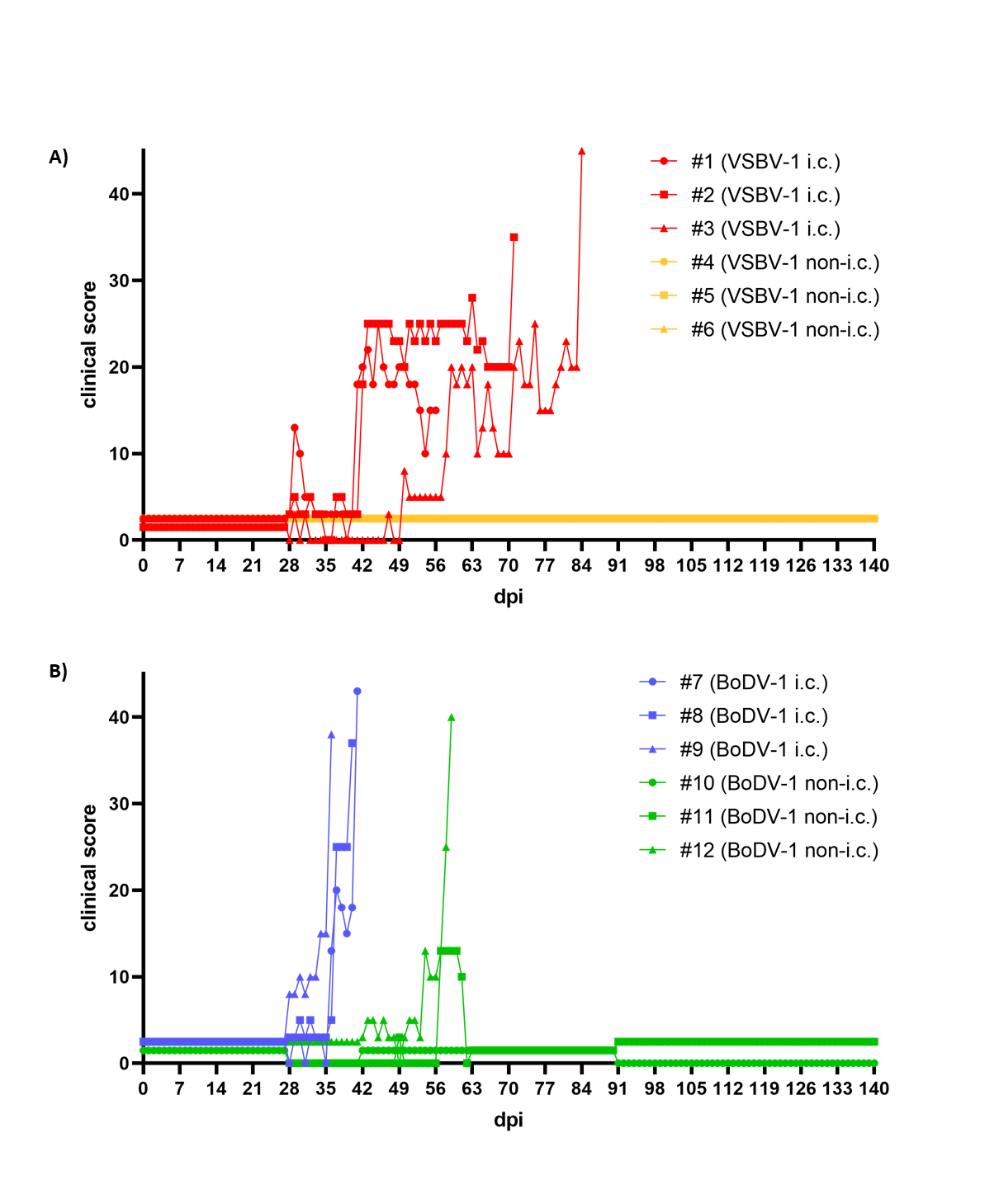


Figure S2: Clinical scores of the twelve rhesus macaques inoculated with A) VSBV-1 and B) BoDV-1. i.c. = intracerebral; non-i.c. = peripheral routes


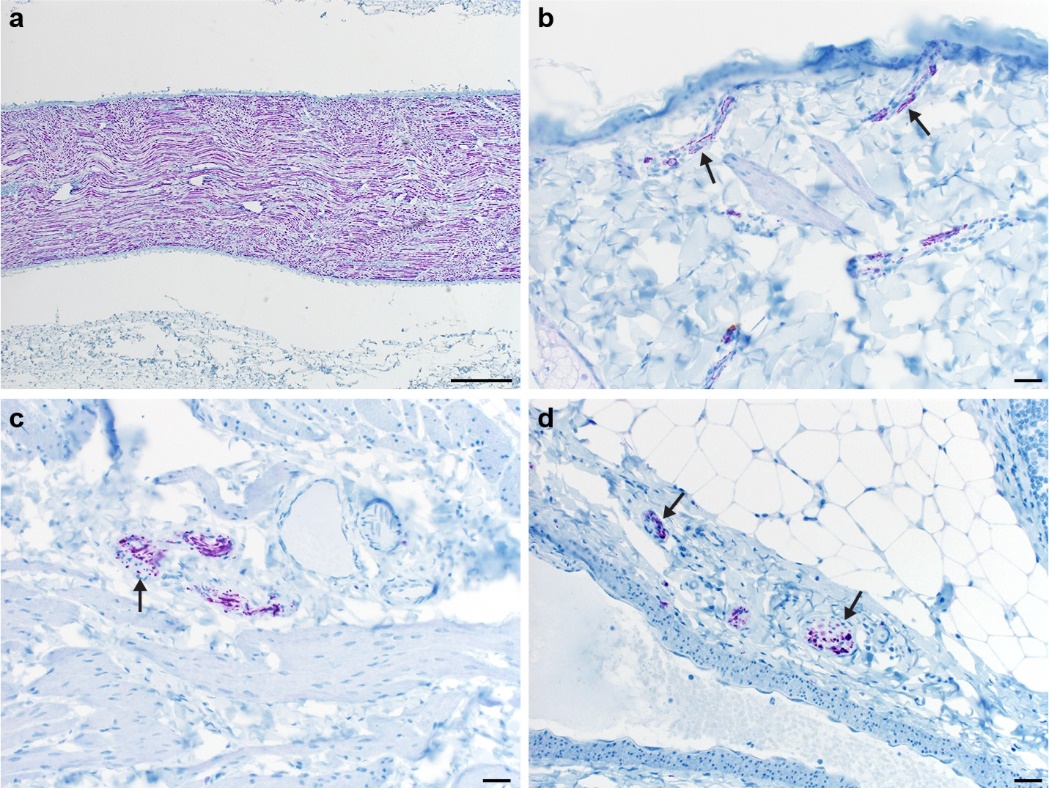


Figure S3: Immunohistochemistry in peripheral nerves infected with BoDV-1 (NHP #12). Immunoreactive peripheral nerve tissue (purple, arrows). A) Brachial plexus - 40X; bar = 200 µm. B) Skin - 200X; bar = 20 µm. C) Urinary bladder - 200X; bar = 20 µm. D) Subcutaneous tissue - 200X; bar = 20 µm.
